# Endometrial Cancer Presenting as Acute Urinary Retention: a Case Report and Review of the Literature

**DOI:** 10.1186/1757-1626-2-9388

**Published:** 2009-12-23

**Authors:** Samer Tannus, Ilan Atlas

**Affiliations:** 1 Department of Obstetrics & Gynecology, The Baruch Padeh Medical Center, Poriya, Lower Galilee 15208, Israel

## Abstract

**Introduction:**

Endometrial cancer is the most common gynecologic malignancy. Most women present with abnormal vaginal bleeding. Cervical stenosis can prevent early uterine bleeding leading to delayed diagnosis of the disease.

**Case presentation:**

A 67 years old Caucasian woman presented with three days history of right flank pain and difficulty in urination. Her medical history included cervical cautery due to ectropion 15 years previously. In physical exam a huge cystic mass that proved to be a large hematocervix was bulging in the vagina. Computed tomography of the abdomen revealed a distended cervix adjacent to distended urinary bladder secondary to endometrial cancer.

**Conclusion:**

To our knowledge this is the first case report describing acute urinary retention secondary to hematocervix. Cervical stenosis is common in elderly postmenopausal women and can prevent early manifestation of endometrial pathology and can be associated with local complications.

## Introduction

Endometrial cancer is the most common gynecologic malignancy and the fourth most common cancer in women [1]. It has a high cure rate as 92 percent of women are detected in stage I and II of the disease and most present with vaginal bleeding as the chief complaint. Cervical stenosis is a common occurrence in elderly women and can prevent early uterine bleeding of endometrial cancer and delays the diagnosis. We describe an unusual manifestation of low grade endometrial cancer diagnosed in advanced stage presenting as acute urinary retention secondary to hematocervix. A case report and review of the literature are presented.

## Case

A 67 years old Caucasian woman gravida 3, para 1 presented with three days history of right flank pain and difficulty in urination. Her medical history was significant for obesity, hypertension and previous salpingectomy due to ectopic pregnancy. 15 years previously a cervical cautery was performed due to cervical ectropion.

On admission her vital signs were stable. Physical examination revealed tenderness in both costovertebral angles and fullness in the lower abdomen. In gynecologic exam a huge soft cystic mass was bulging in the vagina to the level of the hymen. It was suspected to be a distended cervix. The external cervical os was not identified.

**Figure 1:**
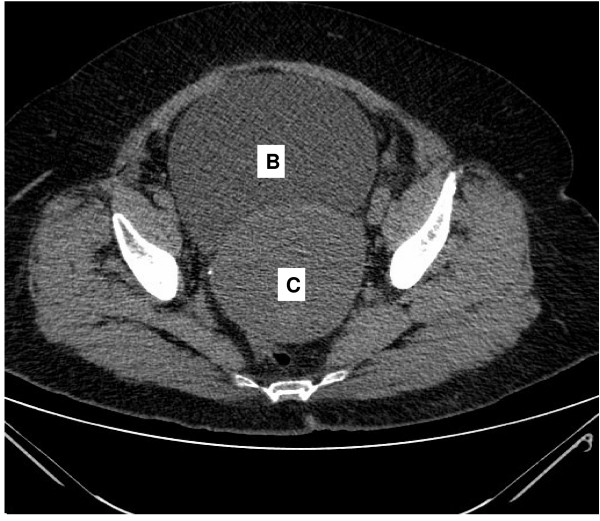
**Ct scan of the abdomen reveals a distended urinary bladder (B) and adjacent distended Hematocervix (C)**.

**Figure 2:**
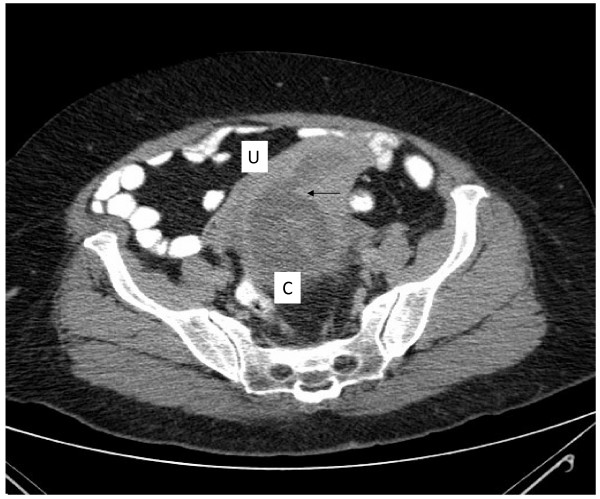
**Ct scan of the abdomen reveals a uterus (U) with dilated cavity (arrow) on top of the dilated cervix (C)**.

Computed tomography of the abdomen revealed a distended urinary bladder with bilateral hydronephrosis and hydroureter. Adjacent to the bladder was a cystic mass of 10 cm in the pelvis that was suspected to be a dilated cervix (figure 1). The uterus was extra pelvic on top of the mass and was of normal size with dilated cavity up to 2.5 cm (figure 2). No ascites or pelvic lymphadenopathy were noticed. Urethral catheterization drained 2000 ml of clear urine. Blood and chemistry profile were normal.

An explorative laparatomy was performed; the uterine cervix was found to be distended with very thin walls and when was incised proved to be a large hematocervix and about 700 ml of old blood was drained. Due to cervical distention and difficulty differentiating the margins of the cervix from the vagina a modified radical hysterectomy was performed. Due to suspected malignancy, surgical staging was completed by omentectomy and lymph node dissection.

Histological examination of the specimens revealed grade I adenocarcinoma of the uterus located in the fundus with more than two thirds myometrial invasion. There was no lymph-vascular space involvement. The lower uterine segment, cervix and omentum were free of disease. Two of the pelvic lymph nodes were positive for metastases; stage IIIC according to the International Federation of Gynecologists and Obstetricians staging system. Following surgery the patient was treated by pelvic radiation and was doing well one year after surgery with no signs of disease recurrence.

## Discussion

Acute urinary retention is unusual and rare manifestation of endometrial cancer. We assume that the previous cervical cautery resulted in cervical stenosis that concealed the uterine bleeding. The continuous blood accumulation distended the cervix and posed direct pressure on the adjacent urethra causing urinary obstruction. Bolton et al [2] described a similar presentation of endometrial cancer causing urinary retention, in that case, previous vaginal cautery resulted in vaginal stenosis and to hematocolpos formation causing pressure on the urinary tract.

Cervical stenosis can lead to delayed detection of endometrial pathology. It is common in postmenopausal women and can result from aging, infection, malignancy, previous surgery or radiation. In our case, cervical distention is the result of continuous uterine fluid accumulation. Uterine fluid collection can be found in approximately 9-12% of asymptomatic, post-menopausal women [3,4] and is commonly associated with cervical stenosis [5]. The significance of intrauterine fluid collection and cervical stenosis was investigated in previous studies with conflicting results; Breckenridge et al [6] investigated women with uterine fluid collection and found malignancy in 16 of 17 such patients, 11 of which had cervical stenosis and the final diagnosis was carcinoma of the uterine corpus or cervix. McCarthy et al [7] reported tow malignancies out of eight patients with asymptomatic intra uterine fluid collection. On the other hand, In a recent prospective study by Debby et al [8]; 82 postmenopausal women with cervical stenosis and endometrial fluid collection were investigated by curettage or hysteroscopy; no patient was found to have an underlying malignancy, 84 percent had an atrophic endometrium and one patient was diagnosed with a complex hyperplasia with atypia of the endometrium, moreover the incidence of intrauterine pathology increased with the increasing thickness of the endometrium as observed in ultrasound with all patients with endometrial thickness less than 3 mm (Two endometrial layers excluding the uterine fluid) had atrophic endometrium. Goldstein [9] investigated 30 women with uterine fluid collection and cervical stenosis; no endometrial pathology was detected when the surrounding endometrial thickness was less than 3 mm (two endometrial layers). In three patients with thickened endometrium; hyperplasia without atypia was diagnosed. He proposed that endometrial sampling is necessary only when endometrial thickness surrounding the fluid collection is more than 3 mm on transvaginal sonography and anything thinner is invariably benign. In our case vaginal sonography was difficult to perform due to the vaginal mass and the abdominal sonography was difficult to perform due to the patient obesity.

Delayed diagnosis of endometrial cancer affects prognosis, Schneider et al [10] described seven cases of endometrial cancer that developed far after cervical amputation, in six patients cervical stenosis prevented early uterine bleeding and when diagnosed four patients where in advanced stage of the disease. In our case although the tumor cells were of grade I, it was already stage IIIC when was diagnosed.

## Conclusion

This case demonstrate an unusual and rare presentation of endometrial cancer causing cervical distension and urinary retention. It is the end stage result of continuous fluid accumulation in the uterus and mainly the cervix. To our knowledge this is the first case describing urinary retention secondary to hematocervix. Uterine fluid demonstration is common in postmenopausal women and is frequently associated with cervical stenosis, such cases must prompt early assessment to rule out underlying malignancy.

## Consent

Written informed consent was obtained from the patient for publication of this case report and accompanying images. A copy of the written consent is available for review by the Editor-in-Chief of this journal.

## Competing interests

The authors declare that they have no competing interests.

## Authors' contributions

ST and IA contributed equally in writing this manuscript. IA Performed the gynecologic surgery. ST and IA followed the patient in the gynecologic oncology unit. All authors read and approved the final manuscript
